# Depression According to ICD-10 Clinical Interview vs. Depression According to the Epidemiologic Studies Depression Scale to Predict Pain Therapy Outcomes

**DOI:** 10.3389/fpsyg.2019.01862

**Published:** 2019-08-20

**Authors:** Sabine Fiegl, Claas Lahmann, Teresa O’Rourke, Thomas Probst, Christoph Pieh

**Affiliations:** ^1^Department for Psychotherapy and Biopsychosocial Health, Danube University Krems, Krems an der Donau, Austria; ^2^Department of Psychosomatic Medicine and Psychotherapy, University Medical Center Freiburg, Freiburg im Breisgau, Germany

**Keywords:** mood disorder, self-assessment, evaluation, disability, interdisciplinary treatment

## Abstract

**Purpose:**

Pain and depression have been shown to have a bidirectional interaction. Although several outcome studies have been conducted, it is still unclear if and how depression influences pain outcome. The current study aims to further clarify this relationship by comparing the predicting value of an interview- and a questionnaire-based assessment of depression.

**Patients and Methods:**

This retrospective study analyzed data of *N* = 496 chronic pain patients who received a multimodal pain management program. Multilevel models were performed with depression as predictor, pain measures as dependent variables, and the respective pain score at baseline as covariate. Depression was measured at baseline with (1) a semi-structured psychiatric interview corresponding to the ICD-10 and (2) the Center for Epidemiologic Studies Depression Scale (CES-D). Pain outcomes were pain intensity assessed with the Numeric Rating Scale (NRS), pain disability measured with the pain disability index (PDI), and affective as well as sensory pain perception assessed with the Pain Perception Scale (PPS-A/PPS-S).

**Results:**

At post-treatment, pain intensity (NRS) was higher in patients with depression. This result emerged for interview- (ICD-10) and questionnaire- (CES-D) based depression. These results were significant after correction for multiple testing as well. Moreover, affective pain perception (PSS-A) at post-treatment was higher in patients with depression. Again, this result emerged for interview- (ICD-10) and questionnaire- (CES-D) based depression but it was not significant anymore after correction for multiple testing. Furthermore, pain disability (PDI) was higher at post-treatment in patients with depression according to the CES-D than in those without CES-D depression and this difference in the PDI did not emerge for interview-based depression. Yet, this difference on the PDI between the CES-D depression group and the CES-D no depression group was not significant anymore after correction for multiple testing.

**Conclusion:**

The hypothesis that how depression is assessed – interview-based corresponding to the ICD-10 or with the CES-D – contributes to the association between depression and pain treatment outcome could not be confirmed. Future research should use more than one interview and questionnaire to assess depression, since our results are limited to the clinical ICD-10 interview and the CES-D.

## Introduction

A meta-analysis of epidemiological studies investigating chronic pain revealed prevalence estimates from 8.7 to 64.4 percent depending on how chronic pain was defined (Steingrímsdóttir et al., 2017). Lifetime prevalence of pain complaints ranges from 24 to 37% (Bair et al., 2003). Additionally, pain is the leading cause of years lived with disability (YLD), having low back pain causing 57.6 million and migraine causing 45.1 million of YLD in 2016 (Vos et al., 2017). Major depression, also one of the five leading factors causing YLD (34.1 million) (Vos et al., 2017), is common among patients with chronic pain (Bener et al., 2013; Stubbs et al., 2017). For instance, a large study evaluating the world mental health surveys of multiple western as well as developing countries showed a pooled odds ratio for depression among pain patients of 2.3 (CI: 2.1, 2.5) (Demyttenaere et al., 2007). Similarly, a recent study revealed significant associations between severe pain and depression in 44 of 47 investigated low- and middle-income countries (Stubbs et al., 2017).

Furthermore, chronic pain patients with comorbid depression cause higher health care costs than chronic pain patients without depression (Rayner et al., 2016). Additionally, depression frequency increases with higher age (Morete et al., 2018). Pain and depression also partly share the same neuronal processes, neurotransmitters, and brain structures (Sheng et al., 2017). For example, monoamine neurotransmitters as well as glutamate have been shown to be critically involved in the development of both pain and depression. Chronic pain also potentially reduces dopamine activity, which in turn is involved in the occurrence of depression (Sheng et al., 2017).

Moreover, medical treatment addressing depression has been shown to affect pain as well (Polatin et al., 2018). Medications reported to have both analgesic and psychotropic effects include SNRIs (serotonin norepinephrine reuptake inhibitors), TCAs (tricyclic antidepressants), and anticonvulsants (Hooten, 2016).

Due to the existing relationship between pain and depression, therapy programs are recommended to be multidisciplinary to address both disorders (Bair et al., 2003). Multidisciplinary pain treatment mostly consists of physiological, psychological, and social factors (bio-psycho-social model) (Gatchel et al., 2014). Physiological components address medication, exercise, surgery, sleep, psychological components address cognitions, emotions, behaviors, attention, social components address healthcare, family, and work. Typically, physicians, nurses, psychologists, physical therapists, and occupational therapists are involved in multidisciplinary pain management programs (Gatchel et al., 2014).

A review of McCracken and Turk (2002) revealed different predictors of the outcome of a behavioral and cognitive-behavioral pain treatment, including depression. However, the results on how depression affects the outcome of chronic pain treatments are ambivalent. While several studies found depression to be associated with a worse outcome (Betrus et al., 1995; Bair et al., 2003; Turner et al., 2007), others found that depression does not predict the outcome (Kerns and Haythornthwaite, 1988; Gureje et al., 2001; Glombiewski et al., 2010; Broderick et al., 2016). One study even reported that depression was associated with a better pain outcome (van der Hulst et al., 2008).

One reason for the diverging results may be the use of different methods to assess depression. Many studies used questionnaires to measure depression (Kerns and Haythornthwaite, 1988; Betrus et al., 1995; Turner et al., 2007; van der Hulst et al., 2008; Glombiewski et al., 2010; Broderick et al., 2016), which have economic advantages in being time-efficient and cost-effective (Stuart et al., 2014). Only a few used interview-based methods to assess depression (Gureje et al., 2001; Bair et al., 2003).

In general, structured interview-based methods reportedly best identify mood disorders (Stuart et al., 2014; Hooten, 2016). Questionnaires however, still show superiority in recognition of depression to physicians’ depression diagnosis (Löwe et al., 2004) and have sensitivity rates between 25 and 100 and specificity rates between 22 and 99 (Löwe et al., 2004; Eaton et al., 2007).

In chronic pain patients, Poole et al. (2009) revealed comparable screening of interview-based depression and depression assessed with a questionnaire.

In summary, depression is a critical psychological aspect of chronic pain. Hence, depression, assessed either via questionnaire or interview, has been well-investigated as a predictor of pain treatment outcome. Although Poole et al. (2009) already compared both assessment methods, they did not include pain treatment outcome. To figure out whether the assessment method affects the connection between depression and pain treatment outcome, we investigated both questionnaire- and interview-based depression measurements in one sample.

The current study re-analyzed data of Pieh et al. (2012) who investigated gender differences in pain outcome after a multimodal pain management program. Pieh et al. (2012) found gender differences to have an influence on pain outcome after the therapy. In particular, pain-related differences in daily life were found to be better in women than in men after the therapy. The current study however, investigates differences between different depression measurements in pain outcome after the multimodal pain management program.

Our two research questions are: Is there a difference in the outcome of a multimodal pain management program (1) between patients with and patients without depression as assessed in interviews as well as (2) between patients with and patients without depression measured with a questionnaire?

The aim of this study is to investigate, if there is a difference between clinical interview-based ICD-10-corresponding depression and questionnaire-based depression according to the Center for Epidemiologic Studies Depression Scale (CES-D) in predicting pain outcomes. Furthermore, it aims at ascertaining whether one assessment method of depression is preferable for future research and clinical practice regarding the impact of depression on chronic pain.

As previous research showed ambivalent results with regard to the predicting value of depression on pain outcomes, we expected that interview- (ICD-10), and questionnaire- (CES-D) based depression differ in predicting pain outcomes.

## Materials and Methods

This is a retrospective study and a re-analysis of the data of Pieh et al. (2012). Data were collected from patients with chronic not malignant pain, treated in the pain clinic in Weiden, Germany, between 2006 and 2010. The study was conducted in accordance with the Declaration of Helsinki and ethical laws were applied. All participants signed a consensus declaration and agreed to the analysis of their anonymous data.

### Depression Measurement

#### Semi-Structured Psychiatric Interview

Clinical diagnosis of depression was ascertained by specialists for psychiatry with a semi-structured psychiatric interview corresponding to the International Statistical Classification of Diseases and Related Health Problems (ICD-10) symptom checklist for mental disorders (Janca et al., 1994). The clinical diagnoses F32, F33, and F34.1 were categorized as existing depression diagnosis. Other clinical diagnoses were categorized as no depression diagnosis.

#### Center for Epidemiologic Studies Depression Scale (CES-D)

In the current study, we used the German version of the CES-D (Radloff, 1977) and accordingly the German cut-off of 22 recommended by Hautzinger (2016). The German CES-D matches the English Center for Epidemiologic Studies Depression Scale (Vilagut et al., 2016), which is often used for the operationalization of depression (Burke et al., 2015). For further analyses, we computed the sum value. The CES-D shows good reliability (Cronbach’s Alpha > 0.90) and validity values (correlation with other self-rating instruments for depressive symptoms between *r* = 0.64 and *r* = 0.88) (Hautzinger, 2016).

According to the different depression measurements, four groups result (clinical interview-based depression: yes/no; CES-D cut-off exceeded; yes/no). However, considering our research questions, we compared participants with and without depression diagnosis for both measurements, respectively.

### Pain Measurement

The following pain measures were administered at the beginning (t0) and end (t1) of the multimodal pain management program.

#### Numeric Rating Scale (NRS)

The Numeric Rating Scale is an often used self-rating instrument to measure pain intensity in chronic pain patients with a scale from 0 (no pain) to 10 (worst imaginable pain) (Joos et al., 1991; Jensen et al., 1999). It refers to the past 4 weeks and rates minimum, average, and maximum pain (Ferraz et al., 1990). In the current study, only the average pain rating was used. The NRS features good external validity in correlations with other pain intensity measurements (with e.g., VAS *r* = 0.94 to *r* = 0.96) (Williamson and Hoggart, 2005; Ferreira-Valente et al., 2011).

#### Pain Disability Index (PDI)

In the current study, the German version of the PDI (Dillmann et al., 1994) was used. This self-rating instrument assesses pain related disabilities on a rating scale from 0 (no disability) to 10 (full disability) in the following areas: recreation, social activity, responsibilities, occupation, self-care, sexual behavior, life support activity, and family/home (Tait et al., 1990). The German version of the PDI shows a good internal consistency (Cronbach’s Alpha 0.88) (Dillmann et al., 1994), reveals a relatively low retest reliability (*r* = 0.44), and validation investigation showed relation to communicative behavior of pain patients (Tait et al., 1990).

#### Pain Perception Scale (PPS)

The Pain Perception Scale (Geissner, 1995) is a German instrument for measurement of both the affective (PPS-A) as well as the sensory (PPS-S) component of subjectively felt pain. Items are scored from 0 to 3 (from not to fully appropriate). The validated instrument shows internal consistency values between 0.72 and 0.92 (Cronbach’s Alpha) (Geissner, 1996).

#### Mainz Pain Staging System (MPSS) (Gerbershagen, 1986)

At baseline, the Mainz Pain Staging System was used to assess the pain chronicity stage of the patients. The MPSS grades pain in terms of four pain-related axes: persistence, spreading, medication, and health care utilization. Stage 1 reflects mild chronicity, stage 2 moderate chronicity, and stage 3 severe chronicity. Construct validity of the MPSS has been shown, for example, by Frettlöh et al. (2003).

### Treatment

Participants completed a multimodal pain management program, which was conducted by psychologists, physicians (a specialist for psychosomatic and psychotherapy, a neurologist and an anesthetist), physical therapists, relaxation therapists, a nutritionist, and a social worker. In accordance with the recommendation of Bair et al. (2003) the multimodal pain management program consisted of treatment of both pain and depression. Over 5 weeks groups of an average of 8 patients participated in an outpatient program (Monday to Friday) consisting of standardized group therapy and individual treatment. The standardized group therapy comprised the CBT-oriented modules acceptance, stabilization, resolving conflicts and strengthening social competency, development of resources, as well as implementation in daily life (altogether 6 h per week). Additionally, the group treatment consisted of relaxation techniques (3.5 h per week autogenic training or progressive muscle relaxation), physical therapy (8 h per week), nutrition advice and social counseling (1 h per week), as well as pain education (2 h per week). The individual treatment contained physical therapy (0.5 h twice a week), doctor’s appointment (0.5 h twice a week), and psychotherapy (1 h per week). In summary, every patient underwent 23.5 h therapy per week.

### Statistics

Statistics were performed with SPSS25. The significance level was set at 0.05 and all statistical tests were performed two-tailed. For descriptive statistics we calculated mean (M), standard deviation (SD), and frequencies (*N*).

To evaluate differences in baseline variables between depressed and not depressed patients, *t*-tests for independent samples and chi-squared tests were calculated. To assess the pre-post effect of the multimodal pain therapy on pain outcomes (PDI, PPS-A, PPS-S, and NRS) in depressed vs. not depressed (CES-D or interview-based) participants, *t*-tests for paired samples were performed. Only patients with complete pre-post assessments were analyzed to examine the pre-post changes (missing data was not imputed for this analysis). Effect sizes (d) were calculated according to the following formula: (Mpre – Mpost)/SDpre. Effect sizes will be interpreted as small *d* ≥ 0.2, medium *d* ≥ 0.5, or high *d* ≥ 0.8 effect.

To assess whether the effect of the multimodal pain therapy is associated with depression (research question 1, clinical interview-based depression; research question 2, depression according to CES-D cut-off), multilevel analyses were conducted with the post-values of PDI, PPS-A, PPS-S, and NRS as outcome variables. The full maximum likelihood method was used. Advantages of multilevel models over traditional methods like analysis of (co-)variance are less assumptions and more flexible handling of missing data. To address research question 1, clinical interview-based depression (yes = 1/no = 0) was entered as dichotomous factor and the pre-treatment scores of the respective pain rating were included as covariate. To address research question 2, the CES-D scores were dichotomized according to the cut-off [above/below cut-off 22 (Hautzinger, 2016)] and this CES-D-based depression variable (yes = 1/no = 0) was entered as dichotomous factor and pre-treatment scores of the respective pain rating as covariate.

Results are reported both without and with Bonferroni correction of the significance level.

## Results

### Sample Description

Of the 496 included patients (254 women) 13 did not complete the treatment due to medical complications. Between 4 (ADS; 0.8% of the total sample) and 9 (PPS-S; 1.8% of the total sample) of the patients did not complete the measures at pre-treatment. Between 39 (NRS; 7.9% of the total sample) and 50 (PDI; 10.1% of the total sample) had missing values in the measures at post-treatment. Table 1 shows the sample description and the comparisons in baseline variables for the sample divided by interview-based depression corresponding to the ICD-10. Table 2 presents the sample description and the comparisons in pre-treatment variables for the sample divided by CES-D depression. Independent from the assessment method of depression (ICD-10 interview-based vs. CES-D questionnaire-based), depressed patients had higher pain chronicity (MPSS), higher pain disability (PDI), as well as higher affective (PPS-A), and sensory (PPS-S) pain at pre-treatment (all *p* < 0.05). Furthermore, the interview-based depression group had a longer pain duration than the no interview-depression group (*p* < 0.05) and this difference did not emerge between the CES-D groups. The CES-D depression group had a higher pain intensity and lower education than the CES-D no depression group (both *p* < 0.05) and these differences did not emerge between the interview-based groups.

**TABLE 1:** Comparisons in pre-treatment variables between depressed and not depressed patients according to clinical interview.

	**Clinical interview**		
	
	**Depressed**	**Not depressed**	**Statistics**
**Gender n (%)**			
Male	144 (47.8)	98 (50.3)	χ^2^(2) = 0.28; *p* = 0.599
Female	157 (52.2)	97 (49.7)	
**Age (years)**			
M (SD)	48.02(±9.44)	49.17(±10.92)	*t*(493) = 1.24; *p* = 0.214
**Pain duration (months)**			
M (SD)	92.67(±84.14)	74.44(±68.64)	*t*(326.15) = −2.25; *p* = 0.025
**MPSS n (%)**			
1 mild pain chronicity	1 (0.3)	11 (5.7)	χ^2^(2) = 33.05; *p* < 0.001
2 moderate pain chronicity	68 (22.7)	76 (39.2)	
3 severe pain chronicity	231 (77.0)	107 (55.2)	
**Education n (%)**			
<9 years	11 (4.7)	2 (1.5)	χ^2^(3) = 3.78; *p* = 0.287
9–10 years	207 (88.1)	124 (91.2)	
11–13 years	12 (5.1)	5 (3.7)	
>13 years	5 (2.1)	5 (3.7)	
**NRS**			
M (SD)	7.05(±1.63)	7.01(±1.78)	*t*(489) = −0.23; *p* = 0.815
**PDI**			
M (SD)	41.40(±13.00)	36.87(±13.63)	*t*(488) = −3.70; *p* < 0.001
**PPS-A**			
M (SD)	42.42(±9.48)	38.95(±8.87)	*t*(489) = −4.06; *p* < 0.001
**PPS-S**			
M (SD)	27.17(±8.96)	24.41(±9.06)	*t*(485) = −3.31; *p* = 0.001

*MPSS, Mainz pain staging system; NRS, numeric rating scale of average pain intensity; PDI, pain disability index; PPS-A, pain perception scale affective; PPS-S, pain perception scale sensory; SD, standard deviation.*

**TABLE 2:** Comparisons in pre-treatment variables between depressed and not depressed patients according to self-assessment questionnaire (CES-D).

	**CES-D**		
	
	**Depressed**	**Not depressed**	**Statistics**
**Gender n (%)**			
Male	159 (48.3)	80 (49.1)	χ^2^(1) = 0.03; *p* = 0.875
Female	170 (51.7)	83 (50.9)	
**Age (years)**			
M (SD)	48.71(±9.29)	48.00(±11.44)	*t*(271.02) = −0.69; *p* = 0.490
**Pain duration (months)**			
M (SD)	90.75(±83.16)	76.99(±70.03)	*t*(363) = −1.57; *p* = 0.118
**MPSS n (%)**			
1 mild pain chronicity	4 (1.2)	8 (4.9)	χ^2^(2) = 27.21; *p* < 0.001
2 moderate pain chronicity	75 (22.9)	68 (41.7)	
3 severe pain chronicity	248 (75.8)	87 (53.4)	
**Education n (%)**			
<9 years	11 (4.5)	2 (1.6)	χ^2^(3) = 8.23; *p* = 0.041
9–10 years	220 (90.2)	110 (87.3)	
11–13 years	10 (4.1)	7 (5.6)	
>13 years	3 (1.2)	7 (5.6)	
**NRS**			
M (SD)	7.19(±1.62)	6.72(±1.80)	*t*(486) = −2.91; *p* = 0.004
**PDI**			
M (SD)	42.49(±12.62)	33.71(±13.12)	*t*(485) = −7.15; *p* < 0.001
**PPS-A**			
M (SD)	43.14(±8.75)	36.93(±9.37)	*t*(486) = −7.22; *p* < 0.001
**PPS-S**			
M (SD)	26.70(±8.94)	24.98(±9.28)	*t*(482) = −1.97; *p* = 0.0498

*CES-D, center for epidemiologic studies depression scale; MPSS, Mainz pain staging system; NRS, numeric rating scale of average pain intensity; PDI, pain disability index; PPS-A, pain perception scale affective; PPS-S, pain perception scale sensory; SD, standard deviation.*

### Pre-Post Outcomes

Table 3 shows the results of the *t*-tests for paired samples evaluating the pre- and post-pain changes in patients with interview-based depression, in patients with no interview-based depression, in patients with CES-D depression, and in patients with no CES-D depression. All pain outcomes improved from pre- to post-treatment in each of these four groups (*p* < 0.001). Effect sizes were large for the NRS, medium for the PDI, and low for the PPS-S in each of the four groups. For the PSS-A, large effect sizes emerged in two groups (no interview-based depression; CES-D depression) and a medium effect size emerged in the other two groups (interview-based depression; no CES-D depression).

**TABLE 3:** Pre-post comparisons.

**Group**	**Outcome**	***N***	**Pre-treatment**	**Post-treatment**	**Statistics**	**Effect size**
Interview-based depression			*M*(*SD*)	*M*(*SD*)		
	PDI	267	41.13 (12.96)	34.08 (14.89)	*t*(266) = 9.74; *p* < 0.001	0.54
	PPS-A	271	42.12 (9.61)	34.90 (10.95)	*t*(270) = 12.42; *p* < 0.001	0.75
	PPS-S	270	26.75 (8.76)	23.26 (7.94)	*t*(269) = 6.82; *p* < 0.001	0.40
	NRS	276	7.02 (1.61)	5.72 (1.88)	*t*(275) = 11.17; *p* < 0.001	0.81
No interview-based depression		*N*	*M*(*SD*)	*M*(*SD*)		
	PDI	177	36.46 (13.74)	28.67 (14.53)	*t*(176) = 9.52; *p* < 0.001	0.57
	PPS-A	177	38.76 (8.90)	30.90 (10.44)	*t*(176) = 10.16; *p* < 0.001	0.88
	PPS-S	177	24.14 (9.01)	21.56 (8.24)	*t*(176) = 4.72; *p* < 0.001	0.29
	NRS	179	6.93 (1.79)	5.13 (1.90)	*t*(178) = 11.78; *p* < 0.001	1.01
CES-D depression		*N*	*M*(*SD*)	*M*(*SD*)		
	PDI	294	42.04 (12.55)	34.92 (14.64)	*t*(293) = 10.57;*p* < 0.001	0.57
	PPS-A	302	42.95 (8.75)	35.42 (10.64)	*t*(301) = 12.94; *p* < 0.001	0.86
	PPS-S	300	26.49 (8.74)	23.31 (7.82)	*t*(299) = 6.73; *p* < 0.001	0.36
	NRS	303	7.19 (1.61)	5.75 (1.91)	*t*(302) = 12.57; *p* < 0.001	0.89
No CES-D depression		*N*	*M*(*SD*)	*M*(*SD*)		
	PDI	147	33.51 (13.48)	25.98 (13.70)	*t*(146) = 8.25; *p* < 0.001	0.56
	PPS-A	143	36.30 (9.44)	29.16 (10.24)	*t*(142) = 9.18; *p* < 0.001	0.76
	PPS-S	144	24.26 (9.16)	21.14 (8.46)	*t*(143) = 4.94; *p* < 0.001	0.34
	NRS	149	6.57 (1.78)	4.99 (1.80)	*t*(148) = 9.77; *p* < 0.001	0.89

*CES-D, centre for epidemiologic studies depression scale; PDI, pain disability index; PPS-A, pain perception scale affective; PPS-S, pain perception scale sensory; NRS, numeric rating scale; SD, standard deviation.*

### Research Question 1: Outcomes Subject to ICD-10 Interview-Based Depression

Results of the multilevel models controlling for the respective pain scale at pre-treatment are presented in Table 4. Before and after Bonferroni correction (*p* < 0.05/4 = *p* < 0.0125), the interview-based depression group scored higher on the NRS at post-treatment than the group with no interview-based depression (*p* = 0.001). The higher scores on the PPS-A at post-treatment for the interview-based depression group compared to the interview-based no depression group were significant before (*p* = 0.028) but not after Bonferroni correction.

**TABLE 4:** Results of the multilevel models evaluating interview-based depression (ICD-10) as predictor of pain outcomes.

	**Estimate**	**SE**	**df**	***t***	***p***
**(1) Outcome: NRS**					
Intercept	2.08	0.36	455	5.79	< 0.001
Interview-based depression	0.55	0.17	455	3.32	0.001
NRS pre-treatment	0.44	0.05	455	9.11	< 0.001
**(2) Outcome: PDI**					
Intercept	1.55	1.65	444	0.94	0.346
Interview-based depression	1.93	1.07	444	1.80	0.073
PDI pre-treatment	0.74	0.04	444	19.00	< 0.001
**(3) Outcome: PPS-A**					
Intercept	7.43	1.92	448	3.87	< 0.001
Interview-based depression	1.97	0.90	448	2.20	0.028
PPS-A pre-treatment	0.61	0.05	448	13.08	< 0.001
**(4) Outcome: PPS-S**					
Intercept	9.29	1.00	447	9.33	< 0.001
Interview-based depression	0.38	0.65	447	0.58	0.561
PPS-S pre-treatment	0.51	0.04	447	14.24	< 0.001

*NRS, numeric rating scale; PDI, pain disability index; PPS-A, pain perception scale affective; PPS-S, pain perception scale sensory; SE, standard error.*

### Research Question 2: Outcomes Subject to Depression According to the CES-D

Results of the multilevel models controlling for the respective pain scale at pre-treatment are presented in Table 5. Before and after Bonferroni correction (*p* < 0.05/4 = *p* < 0.0125), the depressed group according to the CES-D scored higher on the NRS at post-treatment than the group with no depression according to the CES-D (*p* = 0.005). The higher scores on the PDI and the PPS-A at post-treatment for the CES-D depression group compared to the CES-D no depression group were significant before (PDI: *p* = 0.018; PPS-A: *p* = 0.015) but not after Bonferroni correction.

**TABLE 5:** Results of the multilevel models evaluating questionnaire-based depression (CES-D) as predictor of pain outcomes.

	**Estimate**	**SE**	**df**	***t***	***p***
**(1) Outcome: NRS**					
Intercept	2.23	0.35	452	6.32	< 0.001
CES-D depression	0.49	0.18	452	2.80	0.005
NRS pre-treatment	0.42	0.05	452	8.55	< 0.001
**(2) Outcome: PDI**					
Intercept	1.58	1.61	441	0.98	0.329
CES-D depression	2.73	1.15	441	2.38	0.018
PDI pre-treatment	0.73	0.04	441	18.15	< 0.001
**(3) Outcome: PPS-A**					
Intercept	8.03	1.91	445	4.21	< 0.001
CES-D depression	2.39	0.98	445	2.44	0.015
PPS-A pre-treatment	0.58	0.05	445	12.10	< 0.001
**(4) Outcome: PPS-S**					
Intercept	8.92	1.03	444	8.68	< 0.001
CES-D depression	1.05	0.68	444	1.54	0.123
PPS-S pre-treatment	0.50	0.04	444	14.13	< 0.001

*CES-D, centre for epidemiologic studies depression scale; NRS, numeric rating scale; PDI, pain disability index; PPS-A, pain perception scale affective; PPS-S, pain perception scale sensory; SE, standard error.*

## Discussion

In the current study, we investigated the relationship between pre-treatment depression and pain outcomes after a pain treatment in chronic pain patients with regard to the way depression was operationalized (interview-based or questionnaire-based according to the CES-D). For pain outcomes, we investigated multiple pain dimensions (pain intensity, pain disability, affective pain, and sensory pain). The conducted pain treatment was a multimodal pain management program consisting of multiple treatment methods and was conducted by different physical and psychological specialists.

Although average pre-post effect sizes for changes in pain intensity were large, the results of the statistical models showed a difference in pain intensity (NRS) between pain patients with depression and those without depression. Pain intensity was higher at post-treatment in patients with depression. This result emerged for interview- (ICD-10) and questionnaire- (CES-D) based depression. These results were significant after correction for multiple testing as well. Additionally, affective pain perception was higher at post-treatment in patients with depression than in patients without depression. Again, this result emerged for interview- (ICD-10) and questionnaire- (CES-D) based depression but it was not significant anymore after correction for multiple testing. Moreover, pain disability (PDI) was higher at post-treatment in patients with depression according to the CES-D than in those without CES-D depression and this difference on the PDI did not emerge for interview-based depression. Yet, this difference on the PDI between the CES-D depression group and the CES-D no depression group was not significant anymore after correction for multiple testing. The findings for the PPS-A and the PDI are, therefore, not as robust as the results for the NRS. It should be kept in mind that the findings are correlational and should be interpreted with caution. Nevertheless, these results do not support our hypothesis that depression differentially predicts pain outcomes depending on the operationalization of depression. The NRS outcome was predicted by depression before and after correction for multiple testing regardless of how depression was assessed.

In the following paragraph, we embed our results in the literature. Previous studies using an interview-based depression assessment did not report worse pain outcome, but rather no association between baseline depression and pain outcome after pain treatment (Gureje et al., 2001). Studies using questionnaires to assess depressive symptoms on the other hand, found both negative, positive, and no correlation between depression and pain outcome (Kerns and Haythornthwaite, 1988; Betrus et al., 1995; Turner et al., 2007; van der Hulst et al., 2008; Glombiewski et al., 2010; Broderick et al., 2016). One reason for this incongruity may be the use of different depression or pain measurements. Previous studies investigating the association between depression and pain outcome either used the Symptoms Checklist-90-Revised (SCL-90-R) (Betrus et al., 1995; van der Hulst et al., 2008) the Beck Depression Inventory (BDI) (Kerns and Haythornthwaite, 1988; Glombiewski et al., 2010; Broderick et al., 2016), or the Depression Adjective Check List (DACL) (Kerns and Haythornthwaite, 1988). No comparable study investigating this topic used the CES-D. To assess pain, other studies used the multidimensional pain inventory (MPI) (van der Hulst et al., 2008; Broderick et al., 2016), the fear-avoidance beliefs questionnaire (FABQ2), the attribution of chronic pain patients inventory (KAUKON), the coping strategies and pain-related distress questionnaire (FESV) (Glombiewski et al., 2010), the pain rating index (PRI), or the West Haven-Yale Multidimensional Pain Inventory (WHYMPI) (Kerns and Haythornthwaite, 1988). Three studies applied pain intensity scales, like the Visual Analog Scale (VAS), and the PDI (Kerns and Haythornthwaite, 1988; van der Hulst et al., 2008; Glombiewski et al., 2010), which were applied in the current study, as well. Future research should therefore address the different impacts of different measurements more precisely.

Another reason for the incongruity in the results could be differences in the investigated study samples. The reported studies investigated chronic pain for hip and knee osteoarthritis (Broderick et al., 2016), low back pain (van der Hulst et al., 2008; Glombiewski et al., 2010), or female pain patients (Betrus et al., 1995). The current study, on the other hand, investigated a heterogeneous sample of chronic pain patients. A heterogeneous sample of chronic pain patients was also investigated by Kerns and Haythornthwaite (1988) who found no association between questionnaire-based depression and pain treatment outcome. Moreover, the current sample exhibited a wide range of pain duration prior to the study. Hence, future studies should focus on pain type and duration.

As several studies in the past showed, pain catastrophizing is an associated variable in the relationship between pain and depression and may have a mediating role (Sullivan and D’Eon, 1990; Quartana et al., 2009). Due to this relation, Edwards et al. (2011) recommend to address both depression, catastrophizing, and pain experience in multimodal therapeutic programs. Moreover, another study reported an indirect relation between catastrophizing and depression via hope- and helplessness (Hülsebusch et al., 2016). Therefore, future studies should also consider potential mediating variables, like pain catastrophizing, hopelessness, and helplessness, when investigating the relationship between depression and pain outcome.

A main limitation of the study is its correlative design. Therefore, no causal inferences can be drawn and the internal validity of the results is rather low. There were several difference between the depressed and not depressed groups at baseline, which need to be considered as confounders. A randomized controlled trial assigning patients with or without depression to either multimodal pain management treatment or a control condition would have a higher internal validity. However, the study was conducted in routine care, which enhances the external validity of the results. Nevertheless, the results can solely be interpreted with regard to the applied measurements. The other main limitation is the heterogeneous sample size of the compared groups. As they arise from the results of the depression measurements the sample sizes were not influenceable as the study was not experimental.

Another limitation of the current study is that data were analyzed retrospectively. For more accurate conclusions, future studies should rather investigate this topic prospectively. Furthermore, the selection of the assessment methods of depression could be a limiting factor. The current study compared interview- and questionnaire-based measurements of depression. More precisely, we investigated semi-structured psychiatric interviews corresponding to the ICD-10 symptom checklist for mental disorders (Janca et al., 1994) and the German version of the Center for Epidemiologic Studies Depression Scale (Radloff, 1977). Although interview-based methods are recommended in general (Hooten, 2016), the Structured Clinical Interview (SCID) or the Composite International Diagnostic Interview (CIDI) are known as the gold standard in assessing depression (Spitzer et al., 1992; Wittchen, 1994; Haro et al., 2006; Eaton et al., 2007). Therefore, it is recommended for future research to use SCID or CIDI for interview-based assessment of depression.

Different self-report questionnaires to assess depressive symptoms have admittedly comparable sensitivity and specificity values (Hooten, 2016). However, scale-specific metrics of different depression questionnaires are heterogeneous (Wahl et al., 2014) and a meta-analytic review revealed variable effects of different measures (Burke et al., 2015). Thus, it is also recommended for future research to use more than one self-rate measurement for depression in order to compare results in one sample. As Burke et al. (2015) mention the CES-D and the BDI as the mostly used measurements of self-reported depressive symptoms, future research may for instance investigate these two questionnaires. Additionally, Poole et al. (2009) report the BDI to be a good screening method for depression in comparison with the WHO (five) Well Being Index (WBI-5) and the Hospital Anxiety and Depression Scale (HADS), Löwe et al. (2004) in turn show superiority of the PHQ-9. Therefore, the PHQ-9 could also be considered for future research.

Altogether, it would be reasonable to conduct a study in the future which compares several depression questionnaires, for example BDI, CES-D, and PHQ-9, and several clinical interviews based on ICD-10 as well as DSM 5, like SCID or CIDI, within one investigation.

Future studies could also assess the construct of depression more differentiated. Blatt (2004) identified a difference in the development and appearance of two subtypes of depression, the anaclitic and the introjective depression. Addressing the differences of these subtypes, the Anaclitic-Introjective Depression Assessment (AIDA) was developed (Rost et al., 2018). Applying AIDA might prove fruitful in further studies on depression as predictor of pain treatment outcome.

Not having gathered confounding variables could also have a limiting effect in the current study. As a meta-analysis of Burke et al. (2015) revealed anxiety to have a larger impairing effect on pain outcome than depression, future studies should consider anxiety, as well.

The comprehensive assessment of pain, however, can be seen as a strength of the present study.

## Conclusion

The way depression was operationalized did not influence whether depression predicts pain outcomes or not.

## Data Availability

Data regarding this study will not be shared, because clinical data was investigated and we made an agreement with the clinic for not to publish participant data, but only analyses and interpretations.

## Ethics Statement

This study was conducted in accordance with the Declaration of Helsinki and ethical laws were applied. All participants signed a consensus declaration and agreed to the analysis of their anonymous data.

## Author Contributions

SF: drafting the work, substantial contributions to the conception and design of the work, analysis and interpretation of data for the work, final approval of the version to be published, and accountability for all aspects of the work. CL: revising and final approval of the work, substantial contributions to the acquisition of data for the work, and accountability for all aspects of the work. TO: language and final approval, revising the work, interpretation of data, and accountability for all aspects of the work. TP: revising the work critically for important intellectual content, substantial contributions to analysis and interpretation of data for the work, final approval of the version to be published, and accountability for all aspects of the work. CP: revising the work critically for important intellectual content, substantial contributions to the acquisition of data for the work, final approval of the version to be published, and accountability for all aspects of the work.

## Conflict of Interest Statement

The authors declare that the research was conducted in the absence of any commercial or financial relationships that could be construed as a potential conflict of interest.
